# Retained Foreign Bodies in the Facial Region: A Report of Two Cases

**DOI:** 10.1002/ccr3.71876

**Published:** 2026-02-02

**Authors:** Hamed Zartab, Dorian Maghsoodloo, Hanieh Babaei, Delnavaz Jan, Mansour Nassirikashani

**Affiliations:** ^1^ Center for Research and Training in Skin Diseases and Leprosy Tehran University of Medical Sciences Tehran Iran; ^2^ Autoimmune Bullous Diseases Research Center Razi Hospital, Vahdat Eslami Square Tehran Iran

**Keywords:** aesthetic procedures, foreign body, iatrogenic, liposuction, needle fracture

## Abstract

Iatrogenic retained foreign bodies can occur even after minimally invasive procedures like liposuction or dental interventions. Persistent or unexplained cutaneous symptoms should raise clinical suspicion. Early imaging and prompt removal are crucial to prevent complications and ensure patient safety.

## 1 Introduction

Retained surgical objects refer to foreign bodies unintentionally left inside a patient following medical or surgical procedures. Complications from retained foreign bodies (RFBs) can emerge right after the procedure or months or even years later [1]. These RFBs can result in symptoms such as pain, swelling, or discharge, and in some cases may lead to serious complications like bleeding, nerve damage, or infection, which can be life‐threatening [2, 3].

Plain radiography, ultrasonography, computed tomography (CT), and magnetic resonance imaging (MRI) are imaging modalities used to detect surgical objects that cannot be identified through direct visual inspection of the operative field [4].

During oral and cosmetic surgeries, limited visibility, difficult access, small instruments, or inexperience of surgeons may cause materials or tools to break, displace, or be left behind in tissues [3]. While complications due to instrument breakage during surgery are exceedingly rare, they can be easily overlooked, resulting in potentially significant clinical consequences.

Herein, we report two cases of iatrogenic RFBs found in situ in the facial region: one following a dental procedure and the other after submental liposuction.

## 2 Case 1 History and Examination

A 42‐year‐old woman with no significant past medical history underwent submental liposuction and lipotransfer at a local medical center for aesthetic purposes. Ten months later, she presented with a firm band‐like sensation beneath her chin with intermittent vague mild pain and discomfort.

Physical examination revealed a firm, palpable structure in the submental region without overlying skin changes or signs of infection. There was no limitation in neck movement or discomfort while swallowing.

## 3 Case 1 Differential Diagnosis

Based on the patient's history and clinical presentation, possible differential diagnoses included postoperative fibrosis, fat necrosis, calcified hematoma, granuloma, or an RFB secondary to the previous liposuction procedure.

Radiographic evaluation was performed to clarify the etiology.

## 4 Case 1 Conclusion and Results

**FIGURE 1:**
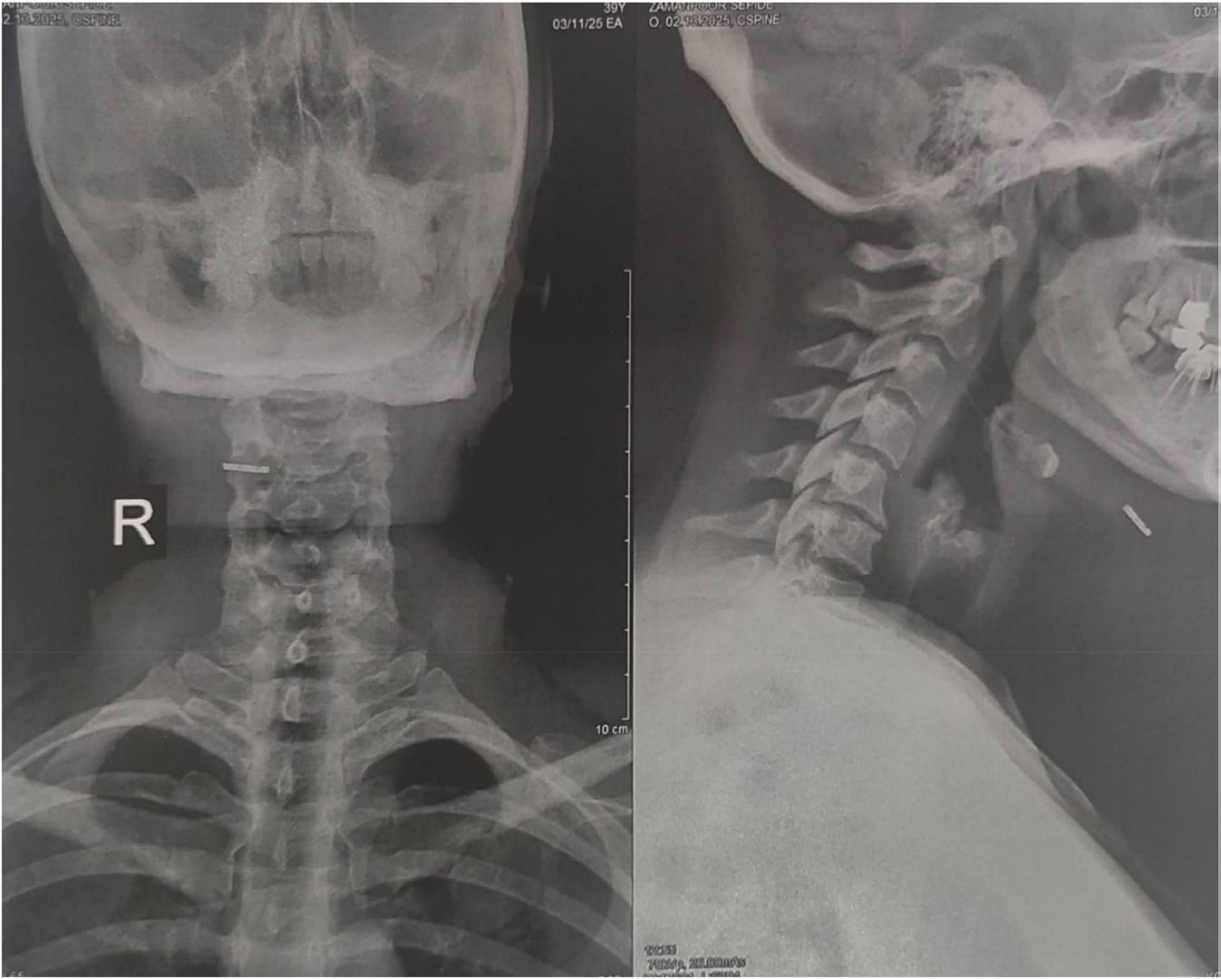
Neck radiography of a 42‐year‐old woman with a broken cannula tip embedded underneath her chin. The broken tip is visible anterior to the hyoid bone. (Left) Antero‐posterior view. (Right) Lateral view.

Neck radiography demonstrated an opaque, linear structure anterior to the hyoid bone on both frontal and lateral views (Figure 1).

**FIGURE 2:**
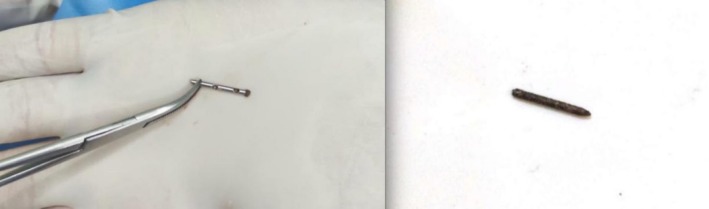
The retained foreign bodies. Left: Broken cannula tip in the patient following liposuction procedure (case 1). Right: Broken needle in the patient with a history of dental procedure (case 2).

Under local anesthesia, the palpable RFB was identified and surgically removed through a small incision. The extracted object was a fractured liposuction cannula tip measuring approximately 2 cm in length (Figure 2, left). Oral cephalexin 500 mg every 8 h was prescribed for the patient. Weekly follow‐up visits were scheduled and completed for 2 weeks. The patient recovered uneventfully, and her symptoms completely resolved during follow‐up. Written informed consent was obtained from the patient and her identity and personal information were kept confidential.

## 5 Case 2 History and Examination

A 65‐year‐old woman presented with an asymptomatic, firm nodule on her left cheek, 2 cm lateral to the oral commissure, persisting for 5 months. She had undergone a dental procedure on the same side of her face about 2 months prior to the development of the nodule. She reported no pain or discomfort on palpation, and her past medical history was nonspecific. Physical examination revealed an asymptomatic, approximately 10 × 20 mm, non‐tender, firm nodule on her left cheek.

## 6 Case 2 Differential Diagnosis

The clinical history and physical examination suggested multiple potential differential diagnoses including post‐traumatic fibrosis, foreign body granuloma, injection granuloma, epidermal inclusion cyst, and retained metallic fragment from broken dental instruments. For further confirmation, ultrasound imaging was performed, which revealed a 17 × 8 × 6 mm dermal collection with a central echogenic 10 mm linear structure, suggestive of a RFB.

## 7 Case 2 Conclusion and Results

Under local anesthesia with Lidocaine, the RFB was explored. An incision on the point closest to the skin surface was made, exposing the foreign body which was then extracted gently with forceps. The excised object was identified as a retained dental needle (Figure 2, Right). The patient had two follow up visits at the end of the first and second weeks and made an uneventful recovery. Written informed consent was obtained from the patient and her identity and personal information were kept confidential.

## 8 Discussion

RFBs are rare but clinically important complications that may occur following any invasive medical procedure. These unwanted events can arise from misplacement of sponges, surgical instruments, or needles. The adverse outcomes vary depending on the nature of the retained object [5]. The two cases presented here illustrate examples of RFBs caused by minimally invasive procedures.

Liposuction is generally considered a safe cosmetic procedure as it involves blunt‐tipped cannulas under manual control [6, 7]. Cannula tip fracture may occur due to multiple reasons, including structural defects, loss of tensile strength, operator technique, and liposuction in fibrous tissue [7, 8]. In the first case, the detection of fractured cannula tip was delayed potentially due to the small size and deep location of the object, as well as mild discomfort, until the patient developed a band‐like sensation. Notably there were no inflammatory reactions or infection, emphasizing that stainless steel RFB are relatively inert [9].

Plain radiography is an inexpensive, easy‐to‐interpret, and reliable imaging modality for detecting metallic RFBs in soft tissues [10]. In the first case, the cannula tip was visible in the neck X‐ray. In the second case, the retained needle following a dental procedure presented as a firm, asymptomatic nodule in the cheek. Such events, though uncommon, have been documented in dental and maxillofacial practice [11]. Possible mechanisms of needle breakage include sudden movement, manufacturing defects, and injection pressure [12]. The absence of clinical symptoms including pain, infection, or inflammatory reactions may complicate early diagnosis. In this case, ultrasonography—as an inexpensive diagnostic imaging modality with no radiation exposure—was effective in identifying the echogenic metallic object within the dermal tissue.

These two cases emphasize several key clinical points. Iatrogenic RFBs are not limited to major surgeries but could be expected following minimally invasive procedures including aesthetic and dental surgeries. Early detection and removal of retained objects are essential to prevent further complications such as fibrosis, infection, migration, or inflammation, which may lead to more severe unwanted events or medicolegal consequences. These incidents underscore the importance of careful inspection of the surgical instruments before and after each invasive procedure. Immediate patient contact and reporting of instrument failure can help prevent further adverse outcomes.

Previous studies have reported RFBs in head and neck soft tissues. Seon et al. reported a case of a broken suture needle embedded in the oral soft tissue during a dental surgery, which was localized using a cone‐beam CT and removed surgically under local anesthesia [3]. The study by Shirol et al. involved a case of a broken cannula tip during a liposuction procedure which remained embedded in the scalp. The fragment was identified through plain radiograph and successfully removed under local anesthesia [7]. Yaqoob et al. reported a case of a periodontal instrument fracture embedded in gingival tissue, successfully removed after periapical radiography [13]. Another recent case report by Mun et al. described surgical removal of a cannula tip embedded in cervical soft tissue after liposuction procedure, emphasizing careful intraoperative instrument management [14]. We presented two cases of RFBs following minimally invasive procedures, diagnosed and removed months after the initial procedure. These cases highlight the importance of clinical suspicion in unexplained cutaneous complaints following invasive procedures, particularly in the absence of a reasonable diagnosis or when the clinical symptom fails to resolve spontaneously or does not respond to appropriate treatment. Preventive strategies including routine evaluation of surgical instruments are crucial for patient safety.

## Author Contributions

**Hamed Zartab:** conceptualization, supervision. **Dorian Maghsoodloo:** writing – original draft, writing – review and editing. **Hanieh Babaei:** writing – original draft, writing – review and editing. **Delnavaz Jan:** writing – original draft, writing – review and editing. **Mansour Nassirikashani:** conceptualization, supervision.

## Funding

The authors have nothing to report.

## Ethics Statement

As a case report with the patient's signed consent, no other ethical review was required.

## Consent

Written informed consent was obtained from both patients included in this case series for publication of their clinical data and any accompanying images.

## Conflicts of Interest

The authors declare no conflicts of interest.

## Data Availability

Data relevant to this case report are included in the article.
